# Removal of Naturally Occurring Strontium by Nanofiltration/Reverse Osmosis from Groundwater

**DOI:** 10.3390/membranes10110321

**Published:** 2020-10-30

**Authors:** Yang-Hui Cai, Xiao Jin Yang, Andrea Iris Schäfer

**Affiliations:** 1 Institute for Advanced Membrane Technology (IAMT), Karlsruhe Institute of Technology (KIT), Hermann-von-Helmholtz-Platz 1, 76344 Eggenstein-Leopoldshafen, Germany; yanghui.cai@partner.kit.edu; 2 Institute of Applied Electrochemistry, Beijing University of Chemical Technology (BUCT), 15 Bei San Huan East Road, Chaoyang District, Beijing 100029, China; yangxj@mail.buct.edu.cn; 3 Department of Water and Environmental Science and Engineering, Nelson Mandela African Institute of Science and Technology (NM-AIST), P.O. Box 447 Arusha, Tanzania

**Keywords:** groundwater treatment, natural organic matter, operating pressure, feed water pH, strontium-organic matter interaction, desalination

## Abstract

Removal of naturally occurring strontium (Sr) from groundwater is vital as excessive exposure may lead to bone growth problems in children. Nanofiltration/reverse osmosis (NF/RO) is commonly used in groundwater treatment due to the high effectiveness and simple maintenance of these pressure driven membrane processes. In this research, a pilot-scale NF/RO system was used to desalinate a natural groundwater sample containing high Sr concentration (10.3 mg/L) and “old” groundwater organic matter (70.9 mg/L) from Esilalei in northern Tanzania to understand the removal of strontium by NF/RO. The impact of applied pressure (10–15 bar) and groundwater pH (3–12) on the membrane performance including permeate flux, strontium and total organic carbon (TOC) flux and removal was investigated. Increasing applied pressure was found to enhance the flux by increasing the driving force and enhance Sr and TOC removal by dilution effect (water flux higher than Sr passage). The alkaline pH caused severe flux decline likely due to membrane fouling and scaling, while it slightly enhanced Sr removal of RO membranes, but weakened the TOC removal. In contrast, acidic and neutral pH of groundwater enhanced TOC removal. These findings suggest that appropriately high applied pressure and acidic pH condition of groundwater are recommended to apply to the NF/RO membrane system in groundwater desalination to achieve better membrane performance.

## 1. Introduction

Strontium (Sr) has become a concern and research interest since radioactive Sr was produced and spread from nuclear weapons or nuclear plants, thus contaminating the environment and biosphere [1,2]. Sr also occurs naturally as the minerals celestite (SrSO_4_) and strontianite (SrCO_3_) [3]. Natural Sr is not radioactive and there are four stable isotopes ^88^Sr (mainly), ^87^Sr, ^86^Sr, and ^84^Sr [3]. The most common radioactive isotope of strontium is ^90^Sr. As a representative radionuclide, ^90^Sr is one of the products of nuclear fission in radioactive waste effluents [4]. It is generated from the reprocessing of nuclear fuels. Radioactive ^90^Sr is very harmful to human health and the environment due to its long half-life (29 years), which is similar to calcium’s contribution in food chains, and its affinity for deposition in plants and bones/skeleton [2,3,5,6]. However, internal exposure to ^90^Sr is linked to bone cancer and soft tissue cancer near the bone, and is suspected of causing leukemia [1,6,7,8]. Non-radioactive strontium has relatively low toxicity, but excessive exposure may lead to bone growth problems in children [4]. Abnormal skeletal development is the primary toxicological effect of excess strontium in animals in the laboratory [4].

Both stable and radioactive Sr can be dissolved in water. Most of the stable Sr input to rivers or groundwater is from the weathering of celestite-rich limestone or rocks, while the radioactive Sr in the water usually originates from nuclear power plants or weapons factories [4,9]. The occurrence of Sr in natural water was ubiquitous with trace concentrations (less than 0.2 mg/L), while some other locations present significant (1–10 mg/L) to high Sr concentrations (more than 10 mg/L) in natural water [10]. For instance, an analysis of 75 samples of major rivers in the USA showed that Sr concentrations ranged from 0.007 to 13.7 mg/L, while groundwater Sr content ranged from 0.2 to 59 mg/L [10]. The maximum concentration was found in a brine well sample (Midland, Michigan) with a Sr content of 2960 mg/L [10].

The WHO guideline value for radioactive Sr is 10 Bq/L, while no WHO guideline value for stable Sr in drinking water was found [11], probably due to limited knowledge about the hazards of stable Sr. United States Environmental Protection Agency (USEPA) set a drinking water health reference level of strontium of 1.5 mg/L and the average annual concentration of ^90^Sr should not exceed 8 pCi/L (0.3 Bq/L) in 2012 [12,13]. Separation and removal of Sr from radioactive wastewaters or natural waters require special attention because it is known to be harmful to human health and the environment and it is likely that not all potential hazards have been identified.

**Figure 1:**
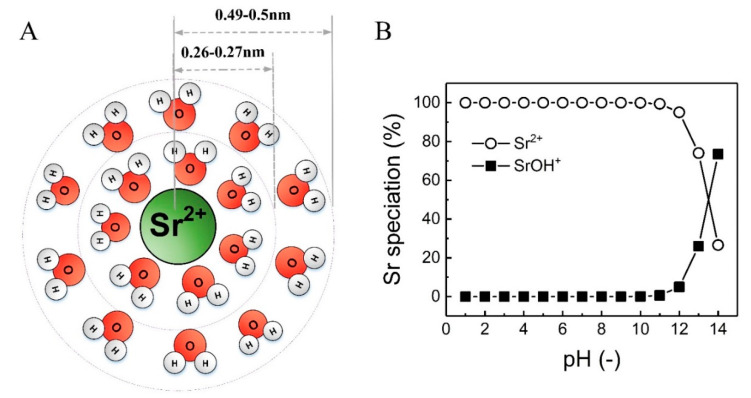
(**A**) Schematic of hydration shells around strontium (Sr^2+^) ion. Inner and outer circles represent the first and second hydration shells; (**B**) basic speciation of strontium in water over a wide pH range (1–14) using Visual MINTEQ software (version 3.1, KTH, Sweden) (1 mg/L strontium chloride (SrCl_2_), typical Sr concentration in surface water [10], 25 °C).

Sr and its salts have chemical properties (ionic radius, hydrated radius, and solubility) similar to calcium (Ca) and barium (Ba), as shown in Table 1. Sr easily loses two electrons and becomes Sr^2+^ in water. When Sr is dissolved in water, the Sr^2+^ ion will hydrate with water molecules and become hydrated Sr^2+^. As shown in Figure 1A, two hydration shells (the first shell with 7–15 water molecules and 0.26−0.27 nm radius, the second shell with 0.49–0.5 nm radius) were reported by Hofer et al. [14]. 

Natural organic matter (NOM) is a complex organic matrix with a wide variety of chemical charges, which is ubiquitous in natural water sources [18]. The major constituents of NOM are humic substances (hydrophobic components). The factors influencing the characteristics of NOM include location, pH, water chemistry, temperature, and biological processes [19]. NOM has a significant impact on many aspects of the water treatment process, including process performance and cleaning [18,20]. NOM can interact with (heavy) metal ions and hence may affect the removal of those ions in membrane filtration through complexation and changes in size and or charge of the solutes [18]. As both Sr speciation and characteristics of NOM can be affected by pH, pH may play an important role in the retention of Sr in the presence of NOM in natural water. As shown in Figure 1B, Sr^2+^ is the main Sr species (100%) in water at pH 2 to 11. Only in very alkaline conditions (pH 12 to 14) does the percentage of Sr^2+^ decrease to 30% and SrOH^+^ increase to 70% and then becomes the main Sr species. These conditions are not to be expected in water treatment applications. Due to the similarities between Sr and Ca, Sr may bind with natural organic matter via chemical interactions (carboxyl and phenolic groups may provide binding sites for multivalent cations) and electrostatic attraction (negative charge of NOM and positive charge of Sr^2+^ ions) [21,22,23]. pH may affect these interactions by changing the charge and structure of NOM [21,24]. Calcium was found to bind with NOM such as humic acid, resulting in a complexation between humic acid and Ca with increasing pH value due to the enhanced negative charge of humic acid [25,26]. Therefore, Sr may behave in a similar manner.

Several physical–chemical separation methods are available to remove Sr from radioactive water or wastewater. For instance, chemical precipitation and flocculation [27], ion exchange [28,29,30,31], adsorption [32,33,34,35], and membrane processes [36,37] have been developed to meet the guideline value of Sr disposal. Among these separation methods, membrane technology has been applied to treat radioactive wastewater (mainly radioactive heavy metal ions) in many nuclear plants around the world due to its high efficiency and relatively simple maintenance [38,39].

Many different membrane technologies can be used for strontium removal from water and wastewater, including electrodialysis (ED), membrane distillation (MD), nanofiltration (NF) and reverse osmosis (RO). Rana et al. [38] have reviewed these membrane technologies and summarized their advantages and limitations. ED is an effective method to separate Sr^2+^ ions (removal 30–99.4% with feed solution 10 mg/L strontium nitrate. By increasing voltage and decreasing flow rate, ED performance can be improved [40]. However, when the solution’s salinity is high (≥ about 3500 mg/L), ED showed a reduced efficiency [41]. Both laboratory and pilot experiments showed that MD is an effective method for Sr removal in radioactive wastewater, especially considering the availability of waste heat in nuclear power plants [42]. With additives (such as chelating micelles, sodium carbonate, activated carbon) in the feed solution enlarging the size of Sr^2+^ ions by sorption, precipitation or complexation, porous microfiltration (MF) and ultrafiltration (UF) membranes can be applied with high flux to remove Sr, even though MF and UF are usually applied as a pretreatment process for nanofiltration/reverse osmosis (NF/RO) and cannot remove dissolved ions such as Sr by itself. However, MF/UF membrane fouling could be significant, and an effective cleaning strategy must be considered. Furthermore, the additives may pass through the membranes into the permeate [36,43,44]. In contrast, dense NF/RO membranes can remove the multivalent Sr^2+^ ions directly [31], while a robust pretreatment process (such as MF/UF) is probably required for the long-term use of NF/RO membranes depending on feed water quality. Membrane characteristics such as charge, pore size or molecular weight cut-off (MWCO) as well as operating conditions will determine the extent of removal that can be achieved, while water quality may also play an important role.

The separation mechanisms of Sr by NF/RO membranes mainly include size (steric), dielectric, and Donnan exclusion (electrostatic repulsion) [45,46,47]. The Sr^2+^ ion is larger than the NF/RO membrane’s MWCO, or pore size, so it can be separated by NF/RO membranes due to size exclusion [46]. The interactions of ions with the bound electric charges induced by ion interfaces between different media dielectric constants cause the dielectric exclusion [48]. While the Donnan exclusion is caused by the electrostatic repulsion between the ions and membrane surface charge [49]. When the membrane surface becomes positive at an acidic pH (below the isoelectric point (IEP) of the membrane), Donnan exclusion will be enhanced, so the retention of Sr is increased [45]. The system hydrodynamics (applied pressure, feed flow velocity, flux, concentration polarization (CP)) and feed water characteristics (pH, salinity, organic matter, etc.) have been reported to also play an important role in Sr retention [45,50,51]. Chen et al. [52] found that Sr retention by NF was more affected by pH and complexation while it was less influenced by the initial Sr concentration. Wadekar and Vidic [46] reported that Sr retention by NF decreased with the increase in pH (due to enhanced charge attraction) and that surface charge effects were the most dominant determining factors. Sr retention by NF increased with the increased feed Sr concentration due to the enhanced Donnan exclusion mechanism [46].

Divalent cations can form complexes with chelating micelles, polymers, and NOM [53], enhancing the Sr removal by more open membranes. On the other hand, fouling problems that are caused by the deposition of such “particles” on the membranes need to be managed. For instance, Cao et al. [44] used sodium carbonate and ferric chloride as precipitating agents to enhance the Sr removal for MF membranes, while significant flux decline was observed due to the fouling. Chen et al. [52] used polypropylene acid (PAA) and disodium ethylene diamine tetraacetate (EDTA-2Na) as a complexing agent to investigate the improvement of Sr removal by NF membranes. It was determined that the PAA and EDTA could improve Sr removal, especially under alkaline conditions. However, the complexation resulted in deposits that caused rapid flux decline and membrane fouling. Cleaning with deionized water and 2% citric acid for 1 h could remove most fouling. Similar conclusions were reported by Hwang et al. [54]. Ding et al. [45] investigated the effect of humic acid (HA) on Sr removal by low-pressure RO. Almost 100% Sr removal and a fast flux decline when filtering Sr and HA were observed, which may be attributed to the formation of Sr-HA complexes, or a gel layer on the membrane surface. The dense fouling layer was observed by SEM. Based on these reports, it can be assumed that strontium may interact with NOM in natural water as well. Therefore, the retention of Sr and NOM could be influenced. However, such interactions between Sr and NOM in natural water have not yet been investigated.

In this study, three different commercial spiral wound NF and RO membranes in a desalination system designed by General Electric (GE) Global Research were used to desalinate natural groundwater containing high strontium concentration and old natural organic matter in the Rift Valley, Esilalei area in Tanzania. The impact of applied pressure and groundwater pH as the main variables on membrane performance (permeate flux, Srretention, and total organic carbon (TOC) retention) were examined. The novelty of this work resides in the fact that this special natural water source (high Sr and old NOM content) is treated by NF/RO membranes for Sr and NOM removal. The main research questions addressed here are (i) is the removal of Sr and organic matter pressure-dependent? (ii) how will groundwater pH variation affect Sr and NOM removal? (iii) which NF/RO membrane is most suitable for this application? The general context of this work is renewable energy powered membrane filtration that is generally aiming at the investigation of energy fluctuations and reducing the specific energy consumption, while the treatment is carefully adapted to water quality.

## 2. Materials and Methods 

### 2.1. Pilot-Scale NF/RO System

**Figure 2:**
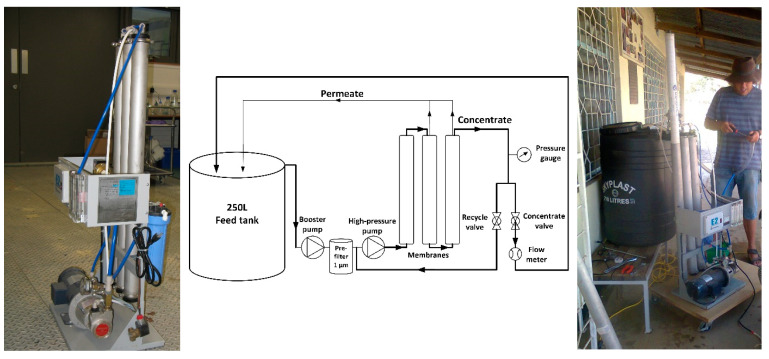
Left: photo of pilot-scale membrane system (GE Osmonics E2-2535TM, photos © Andrea Iris Schäfer) with three 2.5 inch modules in series; middle: schematic of the pilot-scale membrane system; right: set-up of the system at the Ngurdoto Defluoridation Research Station (NDRS) in Tanzania with feed tank.

The pilot-scale membrane filtration system GE Osmonics E2-2535TM (GE Global Research, Munich, Germany) is shown in Figure 2 and was designed for seawater desalination using solar energy. Such a system was previously used by Dialynas et al. [55]. The configuration of the membrane modules is a three-stage single pass using 2.5′’ modules. The concentrate from the last module goes to the next module, and the permeate from each module is collected as a permeate sample. This configuration was specifically chosen, as it can improve the system’s recovery as much as possible, and it is a typical set-up in brackish water desalination even though the last module may perform poorly [56]. The schematic of the pilot-scale membrane system is also shown in Figure 2. 

The system with a direct current (DC) power supply (XHR (100V, 10A), Xantrex, Elkhart, Indiana, USA) consists of a booster pump (model C42D17FK7D, Lesson, Droitwich, UK), a 1 µm pre-filter (Barr + Wray Ltd., SupaGard Filter, Glasgow, UK), a high-pressure pump (series 5, Procon, TN, USA), a flow meter (Hydrasun, Aberdeen, UK), a pressure transducer (Omega Engineering, PGUF-25B-600psi/42bar, Manchester, UK), a thermometer (Omega Engineering, Manchester, UK), three membrane modules and several pipelines. Data are read manually in this system.

The feed water (groundwater as described in Table 4) is initially pumped by the booster pump and high-pressure pump to the membrane modules. Once the operating pressure is higher than the osmotic pressure, the filtration/separation process starts. The operating pressure is controlled by the voltage for the pump and the opening of a needle valve. The system performance as a function of applied power was investigated for the system without membranes (data not included) and a feed flow of 430–613 L/h was achieved at 10 bar. The micro-filter was installed before the high-pressure pump to protect the high-pressure pump and NF/RO membrane from damage caused by large particles.

### 2.2. Membrane Choice

In this research, three 2.5 inch spiral wound NF/RO membrane modules were selected, namely Desal AG-2540 RO membrane supplied by GE Osmonics (Minneapolis, MN, USA), TFC-ULP-2540 RO membrane and TFC-SR2-2540 NF membrane supplied by Koch Membrane Systems (San Diego, CA, USA). The characteristics of the selected membranes are shown in Table 2. The membrane area is 2.6 m^2^ per module. All three membranes are thin-film-composite (TFC) membranes, and the pH range of all membranes is similar. The active layer of Desal-AG and TFC-ULP are made of a full aromatic polyamide, while TFC-SR2 is made of semi-aromatic polyamide [57,58]. The membranes’ support layers consist of a polysulfone midlayer and polyester web support [57]. Both RO membranes Desal-AG and TFC-UPL have a high sodium chloride (NaCl) retention and low permeability, while the TFC-SR2 membrane has the highest permeability but insufficient salt retention and was thus only used in selected experiments with the intent to investigate organic matter removal at low salt retention. TFC-ULP and TFC-SR2 membranes are positively charged at acidic pH (lower than the IEP point, as shown in Table 2) and negatively charged with increasing pH [59,60], while the charge of Desal AG-2540 as a function of pH is not available.

### 2.3. Experimental Protocol

Two sets of experiments, namely applied pressure and pH experiments, were carried out with the three membrane modules to investigate the impact of operating pressure (10–15 bar) and pH (3–12) on the removal of Sr and NOM and their interaction. The detail of the filtration protocol for each experiment is shown in Table 3. Briefly, each experiment includes 5 steps, namely system checking, system start-up, operating pressure setting, sample collection, and system cleaning. The system is designed so that the recovery would be 33–50 % under 10–15 bar for seawater desalination with RO membranes [63]. In this work, the recovery would have been significantly higher at these pressures for the higher permeability membranes. The experiments were carried out at constant pressure, which would have resulted in a recovery variation due to the permeate flow variation since the feed flow was fixed. For pH variation experiments, the system’s recovery was more constant for each membrane, being affected only by flux decline.

The groundwater pH value was adjusted in pH experiments by adding drops of 1 M hydrochloric acid (HCl, Lab Equip Ltd., analytical grade, Dar es Salaam, Tanzania) or 1 M sodium hydroxide (NaOH, Lab Equip Ltd., analytical grade) prior to the experiment.

The operating time was fixed instead of a fixed permeate volume as the permeate and concentrate streams were recirculated. The duration was chosen to achieve a stable condition for a given setting. The experiments were designed to investigate solute retention rather than evaluate energy requirements. The water temperature of each experiment was not controlled, and all the experiments were performed at ambient temperature. The testing location was in Tanzania, which was typically 22 to 29 °C [64]. An adjustment to 20 °C is not meaningful, and the variation over a short-period experiment was not significant compared to other all day experiments [64].

The cleaning protocol after each experiment is summarised as follows: firstly, the low feed flow rate (about 10 L/h) of the cleaning agent was pumped through the membrane system at low pressure (about 0.5 bar) for 15 min. Secondly, the pump was stopped so that the membranes were soaking with the cleaning agent for 2 h. Finally, the system was rinsed by deionized (DI) water for 1 h to remove all the system’s contaminants.

### 2.4. Groundwater Sampling Site and Characteristics of the Water Samples

**Figure 3:**
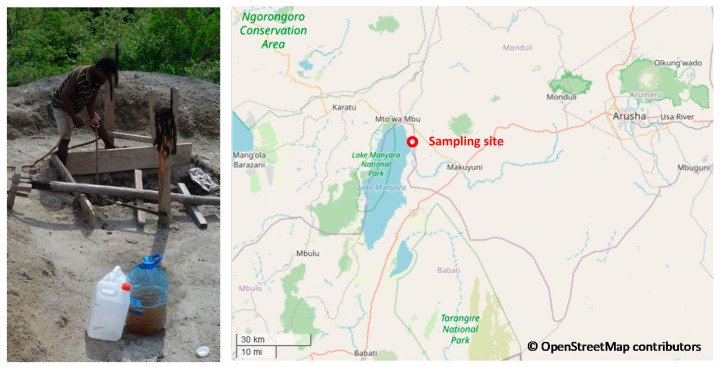
Left: photo of the open well for sampling (© Andrea Iris Schäfer); right: geographical location of groundwater sampling site (Burudika Lodge, Lake Manyara, Tanzania, map © OpenStreetMap contributors under the Open Database License).

The feed groundwater sampling site was at Esilalei nearby the Burudika Lodge, Lake Manyara in northern Tanzania. The exact sampling site on the map and the well are shown in Figure 3. The Global Positioning System (GPS) coordinate of the sampling site is S03°27.684’, E035°54.571’. This site was chosen for this work due to the high concentration of Sr and natural organic matter content following an extensive study of water quality in the region. The site was sampled on several occasions over a year and the water quality of all the samples taken from this site is shown in Table 4. The sample collection involved several wells in the area of which sample five was chosen for treatment investigations. The pH of groundwater sample five was adjusted by HCl and NaOH as described in the experimental protocol section before the pH experiments. 

As shown in Table 4, the groundwater samples in this area contained a high concentration of Sr (1.2–10 mg/L) and a high concentration of natural organic matter (46.6–107 mg/L as total organic carbon (TOC)). The high electrical conductivity (EC, 14,340–28,500 μS/cm) of the samples means high total dissolved solids (TDS) of 7170–14,250 mg/L (estimated from EC values by a conversion factor 0.5) [11,65]. This means that the NF membrane will not be suitable to desalinate this water to drinking water quality. However, a NF membrane (TFC-SR2) was chosen to identify the involved mechanisms when salt and TOC affect Sr transport through solute interactions. This is easier when the retention of organic matter and salt can be separated.

### 2.5. Analytical Methods

The permeate and feed Sr concentrations were analyzed using inductively coupled plasma mass spectrometry (ICP-MS, Agilent Technologies, Agilent 7500). For accurate trace-level Sr analysis, the helium mode was used. The Sr detection processes included filtration of feed samples (to remove large particles), preparation of standard samples and experimental samples (acidification with nitric acid, and measurement by ICP-MS. Total organic carbon (TOC) in permeate and feed was measured by a TOC analyzer (GE Analytical Instruments, now SUEZ Water Technologies & Solutions, Sievers 900) to quantify NOM concentration at the Ngurdoto Defluoridation Research Station (NDRS) where the work was conducted. Liquid chromatography–organic carbon detection (LC-OCD, Model 9, DOC-Labor Dr. Huber, Karlsruhe, Germany) was used to analyze the organic matter fraction in the raw groundwater sample. This analysis was carried out at the Institute of Advanced Membrane Technology (IAMT, KIT, Karlsruhe, Germany) with a sample stored from 2012 until 2020 at 4 °C. Given that the groundwater was collected from an open well, it is assumed that the sample’s storage will not affect its characteristics (organic matter in the groundwater has been degraded in situ and oxygen was not altered through sampling).

The flux *J* (L/m^2^h) is calculated by Equation (1):

(1) $$J=\frac{Q}{A}$$

where *Q* is the permeate flow rate, L/h; *A* is the membrane area, m^2^.

The solute (Sr, TOC) retention (*R*, %) is calculated by Equation (2):

(2) $$R,\;\%=\left(1-\frac{C_{p}}{C_{f}}\right)\cdot 100$$

where $C_{p}$ is the solute permeate concentration, µg/L for Sr and mg/L for TOC; $C_{f}$ is the solute feed concentration, µg/L for Sr and mg/L for TOC.

The solute (Sr, TOC) flux (µg/m^2^h for Sr, mg/m^2^h for TOC) is calculated by Equation (3):

(3) $$J_{solute}=J\cdot C_{p}$$

The absolute error of the solute retention is calculated by Equation (4), according to [66]:

(4) $$\Delta R=\left(100\%-R\right)\cdot \sqrt{{\left(\frac{\Delta C_{p}}{C_{p}}\right)}^{2}+{\left(\frac{\Delta C_{f}}{C_{f}}\right)}^{2}}$$

where *R* is the retention of solute retention, %; $\Delta C_{p}/C_{p}$ and $\Delta C_{f}/C_{f}$ are the relative error of the permeate and feed concentration of solute, respectively. This can be calculated by the relationship between relative error and measured concentration based on the calibration of ICP-MS and TOC analyzer [67]. The analytical error of equipment used in this research was assumed to be similar to equivalent instrument in the IAMT lab (ICP-MS, Agilent 7900, Santa Clara, CA, USA, and SUEZ Water Technologies & Solutions, Sievers 900, Trevose, PA, USA) as these data are not available.

The chemical forms of strontium in real groundwater samples at different pH (3−12) were calculated by Visual MINTEQ chemical equilibrium model software (version 3.1, KTH, Sweden). The dissolved organic matter (DOC) of humic acid from a non-ideal competitive adsorption model combined with the Donnan-type model (NICA-Donnan model) [22] in the software was used as NOM. All samples were used for the speciation to investigate possible variation with water quality.

## 3. Results and Discussion

### 3.1. Impact of Applied Pressure on Permeate Flux

**Figure 4:**
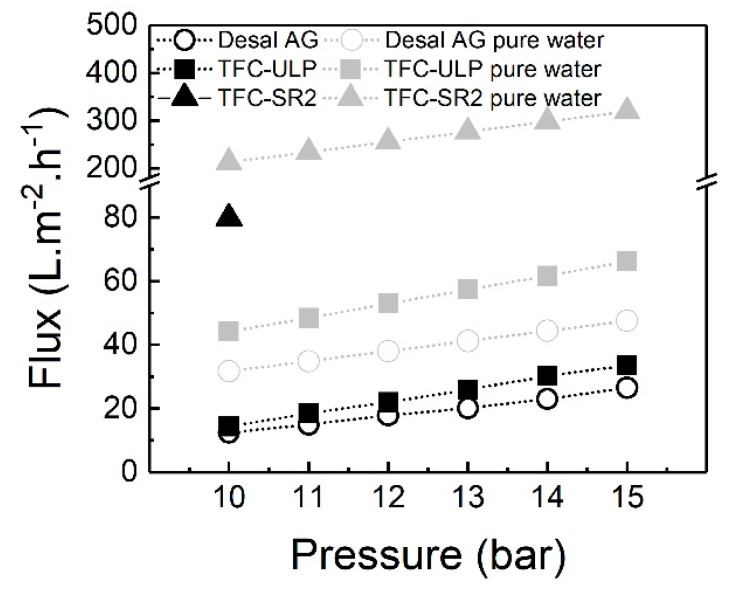
Permeate flux of membranes as a function of applied pressure (10–15 bar) with groundwater sample 5, the pH was about 9, data adapted from [69]; light grey data points are pure water flux of membranes based on the pure water permeability in Table 2.

The impact of different applied pressures on the permeate flux when desalinating real groundwater was investigated at a pressure range of 10–15 bar, as this is a typical operating pressure range for NF/RO groundwater desalination [68]. Results of the Desal AG, TFC-ULP and TFC-SR2 membranes are presented in Figure 4. Due to the high permeability of the TFC-SR2 membrane and its low ion retention, the highest achievable pressure is 10 bar. As a result, only one data point is available, and an operation at such high flux and inevitable high recovery makes operationally no sense.

As shown in Figure 4, each membrane’s permeate flux increased linearly with increasing pressure from 10 to 15 bar as expected, due to increased driving force (the difference between applied pressure and osmotic pressure). The osmotic pressure of sample five is about 6.5 bar, and no flux decline due to fouling or scaling was apparent at higher pressures during the short period of the experiments. Osmotic pressure in the boundary layer would be higher than in the feed solution and increase with salt retention and recovery. It was included in the measured flux. A similar result of a flux (20–100 L/m^2^h) increasing linearly with an increase in operating pressure (2–6 bar) by different NF membranes (XN45, NF90, and NF270) was reported by Chen et al. [52]. For this reason, the pure water flux of each membrane is higher than the flux with real groundwater at different applied pressures due to its higher effective driving force (no osmotic pressure).

Membrane permeability with real groundwater was obtained from the slope of the linear fitting curve, as shown in Figure 4, by considering the osmotic pressure, according to Equation (5). The permeability of TFC-ULP (3.9 L/m^2^h·bar) was calculated, which is higher than the permeability of Desal AG (2.8 L/m^2^h·bar). As can be seen in Equation (5), the flux is determined by the intrinsic membrane permeability, the applied pressure and the osmotic pressure of the feed solution. When the same feed water is applied and operating pressure is fixed, the flux will be determined by the membrane permeability. Due to permeability of TFC-SR2 > TFC-ULP > Desal AG, the flux is TFC-SR2 > TFC-ULP > Desal AG at the same pressure (10 bar, see Figure 4).

(5) $$J_{V}=L_{P}\left(\Delta P-\sigma \Delta \pi \right)$$

where $J_{V}$ is the permeate flux, L/m^2^h; ΔP is the pressure difference and Δπ is the osmotic pressure difference between the feedwater and the permeate, bar; σ is the reflection coefficient, a measure of the selectivity of the membrane, which can be derived from steady-state permeate measurements, 0 ≤  σ ≤ 1 [70]; $L_{P}$ is the permeability (L/m^2^h·bar).

In summary, the permeate flux of the two membranes increased linearly with the increase in applied pressure (10–15 bar), and the TFC-ULP membrane had a higher flux than the Desal AG membrane due to its higher permeability. TFC-SR2 had very high flux due to its much high permeability (see Table 2), and the filtration at higher pressure (> 10 bar) is not meaningful due to extremely high recovery. In the next section, the impact of increasing applied pressure on strontium removal with groundwater will be discussed.

### 3.2. Impact of Applied Pressure on Sr Removal With Groundwater

**Figure 5:**
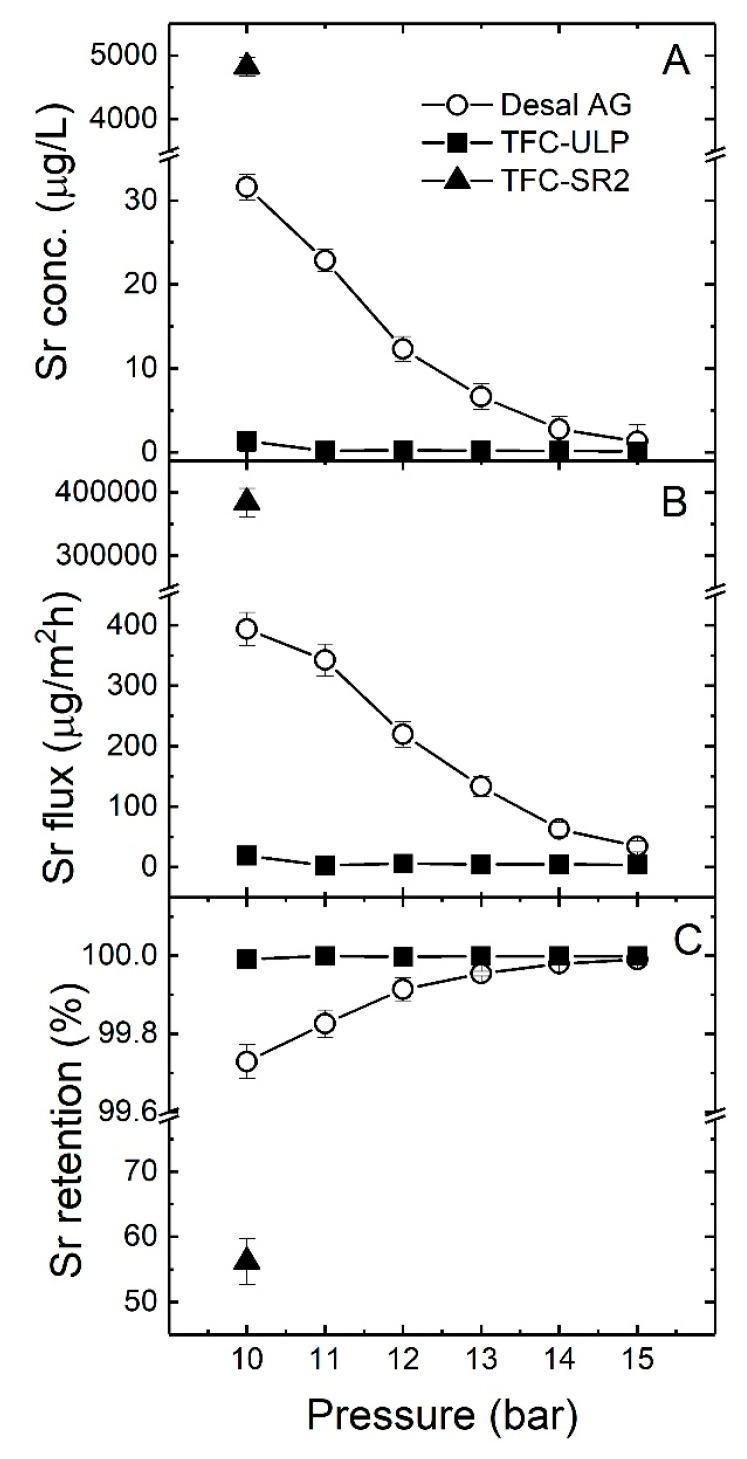
(**A**) Sr permeate concentration, (**B**) Sr flux and (**C**) strontium retention as a function of applied pressure (10–15 bar) with groundwater sample 5 (feed Sr concentration 10.3 mg/L). The pH was about 9, data adapted from [69].

The impact of increasing applied pressure on Sr removal with groundwater by different membranes was investigated by applying the pressure of 10–15 bar on the system. The Sr retention at each applied pressure was determined. Sr permeate concentration, Sr flux and retention are presented in Figure 5.

As shown in Figure 5A–C, Sr removal was very high (99.7–100%) for TFC-ULP and the Desal membranes. Sr permeate reached a concentration of < 40 µg/L from a feed concentration of 10.3 mg/L with the denser RO membranes, while for loose NF membrane TFC-SR2, the Sr permeate concentration reached 4832 µg/L and low Sr removal (56%). The Sr permeate concentration of the TFC-ULP was always lower than that of the Desal AG, which is consistent with the flux performance. It means TFC-ULP is capable of a better Sr removal. As shown in Figure 5A,B, the Sr permeate concentration and Sr flux decreased sharply with increasing applied pressure for the Desal AG membrane, while this effect was negligible for the TFC-ULP membrane. This result is consistent with the findings of Bergman [68], where the salt permeate concentration decreased with an increase in applied pressure. Due to the RO membrane pore size being smaller than the size of hydrated Sr, the steric retention mechanism plays an important role in Sr retention (see Table 1 and Table 2). Thus, even though an increase in operating pressure enhanced the CP, the Sr retention still increased. As shown in Figure 5C, the Sr retention of the Desal AG membranes increased marginally with applied pressure, while that of TFC-ULP remained very high (>99.5%) throughout different pressures. It appears that the enhancement of Sr removal increased with pressure for the Desal AG membrane. This could be explained by the dilution effect of permeate flux where the increase in permeate flux is higher than the increase in Sr passing through the Desal AG membrane by comparing Figure 4 and Figure 5B. 

In summary, an increase in applied pressure (10–15 bar) enhanced the Sr removal of Desal AG, while this effect was negligible for TFC-ULP since it had a very high Sr retention. TFC-SR2 had relatively low Sr retention, probably due to its large pore size. In the next section, the impact of applied pressure on TOC removal will be investigated.

### 3.3. Impact of Applied Pressure on TOC Removal

**Figure 6:**
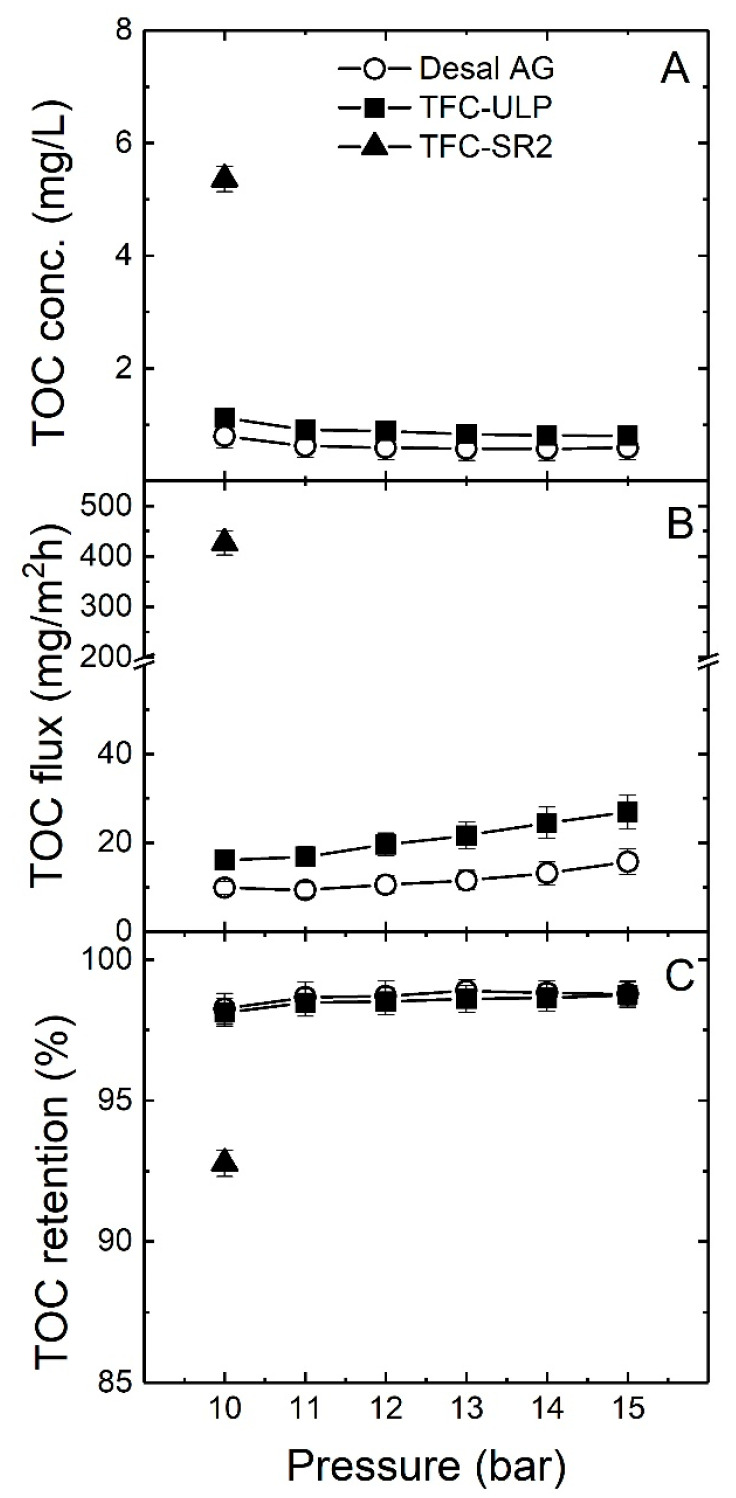
(**A**) Total organic carbon (TOC) permeate concentration, (**B**) TOC flux and (**C**) TOC retention as a function of applied pressure (10–15 bar) with groundwater sample 5 (feed TOC concentration 70.9 mg/L). The pH was about 9, data adapted from [69].

The TOC permeate concentration, TOC flux and TOC removal as a function of applied pressure (10–15 bar) are presented in Figure 6. 

As shown in Figure 6A–C, TOC removal of two RO membranes was high (98–99%), given those permeate concentrations of about 1 mg/L are achieved from a feed concentration of 70.9 mg/L. For the TFC-SR2 NF membrane, TOC removal was about 93%. When we increased the applied pressure (10–15 bar), the TOC permeate concentration of both RO membranes decreased. This can be explained by the fact that the increase in flux is much higher than the increase in permeate TOC flux with pressure. The TOC flux increased with pressure, probably because a fraction of the TOC permeate concentration (low molecular weight (LMW) organics) increases with recovery at the membrane surface so that the diffusion of LMW organics was enhanced. 

The TFC-SR2 membrane had very high TOC flux and TOC permeate concentration as expected. The Desal AG had a lower TOC permeate concentration than TFC-ULP, which means Desal AG had better TOC removal than that of TFC-ULP membrane. Moreover, starting from 11 bar to 15 bar, the decrease in TOC permeate concentration and the increase in TOC retention became stable with the increase in applied pressure, indicating that the primary removal mechanism is size exclusion. While TOC is expected to be entirely removed by RO membranes, the results from Figure 6 show that about 1.5% of organic matter could pass through the RO membranes. This fraction may be the low molecular weight organic matter in the groundwater that cannot be entirely removed by RO membranes. This could be explained well by comparing the LC-OCD results between feed and permeate samples. While the water samples from Tanzania were not sent for LC-OCD analysis and no instrument was available on site, samples from different experiments that were sent for LC-OCD analysis had shown significant regrowth and had to be repeated in a laboratory environment [71]. This is why LC-OCD analysis was performed in a controlled laboratory study at a later date.

**Figure 7:**
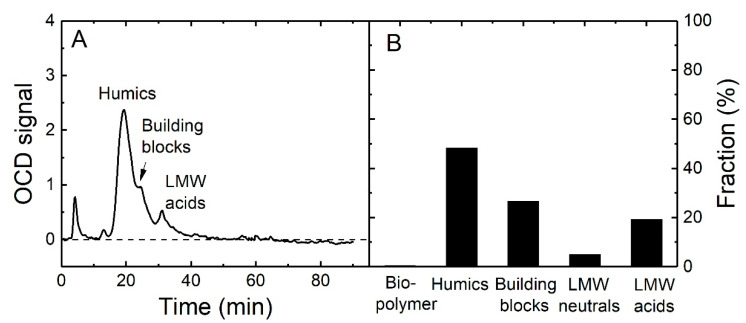
(**A**) Liquid chromatography–organic carbon detection (LC-OCD) signal of groundwater sample 1 as a function of time; (**B**) organic matter fraction in groundwater sample 1 (from Eslalei Lodge, water quality as shown in Table 4).

Therefore, the LC-OCD analysis of the groundwater sample was carried out to determine the content of the LMW organics to confirm the hypothesis. The results are presented in Figure 7. As shown in Figure 7B, the main organic matter composition of the groundwater sample in the Esilalei Lodge area are humic substances (50%), building blocks (25%), and low molecular weight (LMW) neutrals and acids (total 25%) while the fractions of bio-polymers are negligible. This indeed indicates a high LMW fraction. This result is likely due to the ancient nature of the groundwater in the region and a lot of the organic matter has been degraded in the aquifer with time and may not be replenished in this region [72]. This would explain the high contribution of smaller fractions. 

Large organic compounds, such as biopolymers and humic substances, can be totally removed by NF and RO membranes due to their large size, while the LMW neutrals and acids are more challenging to remove [61]. Schäfer et al. [61] found that most LMW acids could pass through NF membranes and cause their lower rejection. Yoon and Lueptow [73] determined that smaller LMW organic compounds had a lower rejection (<10–20%) by NF/RO membranes, while Meylan et al. [74] identified a large portion of LMW organic compounds (especially LMW neutrals) from lake water still present in NF membrane permeate. All the results confirm that the 1.5% of TOC that passes through the RO membranes is most likely due to the relatively large amount of LMW organic matter in this groundwater. Such permeation of organic compounds can be the source of significant regrowth in the permeate if the concentration of LMW organics in source waters are high, as has been observed in studies using some of the tropical blackwaters in Tanzania [72,75].

In summary, an increase in applied pressure (10–15 bar) enhanced the TOC removal for both RO membranes, while the Desal AG membrane had slightly better TOC removal than TFC-ULP. For TFC-SR2, high TOC permeate concentration and TOC flux was observed, which was probably due to the large pore size so that the LMW organics could pass through the NF membrane. LC-OCD results of the groundwater sample supported the predominance of LMW acids in groundwater, which probably led to only 98.5% TOC removal by RO membranes.

In the next section, the impact of groundwater pH on strontium speciation will be analyzed, as this is expected to affect retention and organic matter interaction.

### 3.4. Impact of Groundwater pH on Sr Speciation

**Figure 8:**
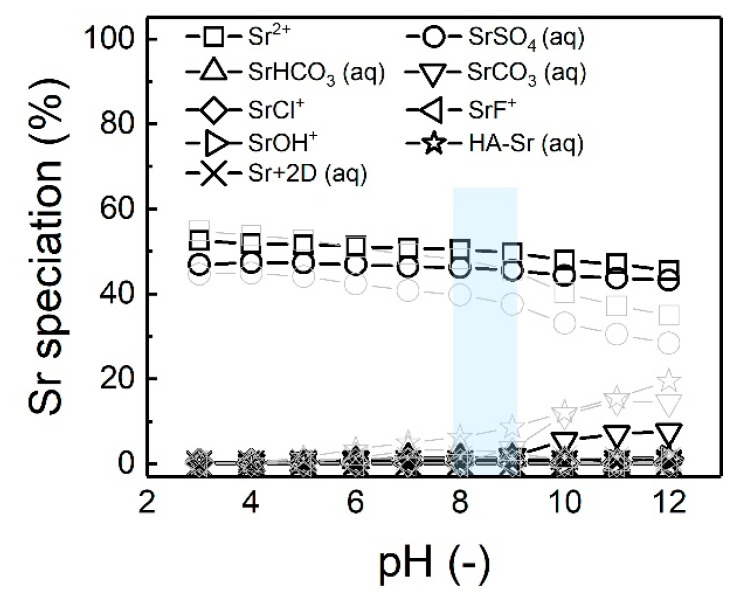
Strontium speciation in groundwater sample 5 and simple 1 as a function of pH (3–12), which was simulated and obtained by Visual MINTEQ. The light blue area represents the pH range of groundwater samples 1–5; light grey data points are Sr speciation in sample 1 as a function of pH.

Water quality, particularly pH and ionic composition of water, plays an important role in Sr’s chemical forms, which will affect the Sr retention of membranes. To investigate the impact of groundwater pH on Sr speciation, the water quality parameters of groundwater samples 1–5 (see Table 4) with different pH values (3–12) were input into Visual MINTEQ software (version 3.1, KTH, Sweden). Sr speciation of groundwater sample five and sample one as a function of pH is presented in Figure 8, since sample five is used for the experiments and sample one had the largest difference with sample five.

As shown in Figure 8, Sr^2+^ ions (46–53%) and dissolved SrSO_4_ (42–47%) are the primary chemical forms of Sr in groundwater sample five in the pH range 3–12, even though their concentrations decrease with increasing pH. When the pH is higher than eight, the dissolved SrCO_3_ concentration increases significantly up to 9%, which increased the tendency of saturated SrCO_3_ due to its very low solubility (about 6 mg/L, see Table 1). This increases the SrCO_3_ scaling potential during the membrane process at high pH. However, compared to the calcium carbonate (CaCO_3_) scaling potential, it is less significant because the calcium ion (Ca^2+^) concentration (210.5 mg/L, see Table 4) is much higher than the Sr^2+^ concentration (10.9 mg/L) even though the solubility of CaCO_3_ is higher (about 13 mg/L). 

When water quality changes, as is the case for the different samples (data in grey), the Sr^2+^ and dissolved SrSO_4_ become significantly less, while the HA-Sr complex becomes more significant at high pH with sample one. Sample one has a lower Sr concentration (1.2 mg/L Sr) than sample five (10.3 mg/L Sr). This demonstrates that groundwater composition (water chemistry) plays an important role in the Sr speciation, which can change with time. Such changes will impact treatability.

In summary, Sr^2+^ ion and dissolved SrSO_4_ are the primary chemical forms of Sr in this groundwater sample in the pH range 3–12. At high pH (>10), the dissolved SrCO_3_ becomes the second main Sr chemical form, which increases the SrCO_3_ scaling potential. However, SrCO_3_ scaling is less significant than CaCO_3_ scaling because concentrations of Sr tend to be lower. In the next section, the impact of groundwater pH on the permeate flux will be analyzed, followed by retention behavior. 

### 3.5. Impact of Groundwater pH on Permeate Flux

**Figure 9:**
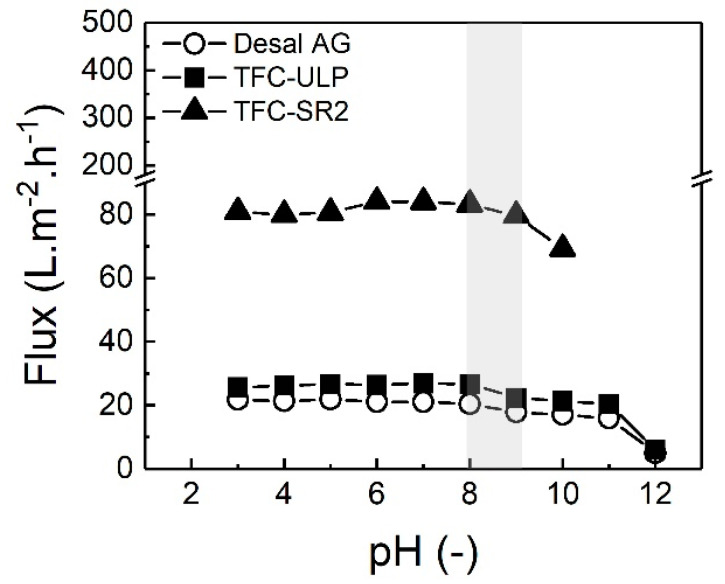
Permeate flux of three membranes as a function of pH (3–12) with groundwater sample 5. The grey area represents the pH range of groundwater 1–5. The applied pressure was 10 bar, data adapted from [69].

The impact of groundwater pH on permeate flux by different membranes was investigated by adjusting the different pH (3–12) of groundwater prior to filtration experiments. The applied pressure was 10 bar as this is the typical operating pressure of NF/RO membranes for brackish water desalination and also is the maximum operating pressure for the TFC-SR2 membrane, which has a higher permeability. The permeate flux as a function of groundwater pH is presented in Figure 9.

As shown in Figure 9, in acid and neutral conditions of groundwater (pH three to eight), the permeate flux of the three membranes was relatively constant. Similar results were observed by Chen et al. [52]. When pH was higher than eight, permeate flux of all membranes started to decrease with increasing pH. Schäfer et al. [26] investigated the effect of divalent cations concentration (Ca^2+^) on NF’s NOM fouling. They found that the irreversible fouling occurred at high calcium concentrations, due to the highly compactable floc-like structure of Ca-humate complexes. Therefore, it is likely that the CP causes gel formation and enhances solute-solute interactions such as the formation of humic–ionic bridges or complexes (such as Ca/Sr-humate complex). Interaction between Ca/Sr and NOM and such gel formation will cause flux decline and potentially fouling during longer-term operation. At high pH, NOM’s negative charge may be further enhanced [71], thus probably increasing the interaction between Ca/Sr and NOM. 

TFC-SR2, as the NF membrane had the highest permeate flux due to its highest permeability (see Table 2), showed the strongest flux decline from pH nine. When the pH was higher than 10, the filtration experiment had to be suspended due to the TFC-SR2 membrane’s fouling. The permeate flux of RO membranes (Desal AG and TFC-ULP) decreased strongly only when the pH increased to 12. The results suggest that NF may cause more severe flux decline than RO membranes when the operating time was fixed because of the very high recovery at such high flux. This flux decline is probably due to the thicker CP layer (much higher permeability) of TFC-SR2 and pore blockage for the more open NF membrane by humate-ionic complexes and/or scaling (CaCO_3_, SrCO_3_, as discussed in Section 3.4) than the tighter RO membranes [26].

In summary, the permeate flux was not affected at pH 3–8, while the flux started to decrease, probably due to membrane fouling, when the pH of groundwater was higher than eight. The TFC-SR2 NF membrane caused more flux decline than RO membranes at high pH when filtration time was fixed probably due to higher CP. In the next section, the impact of groundwater pH on strontium removal will be analyzed.

### 3.6. Impact of Groundwater pH on Strontium Removal

**Figure 10:**
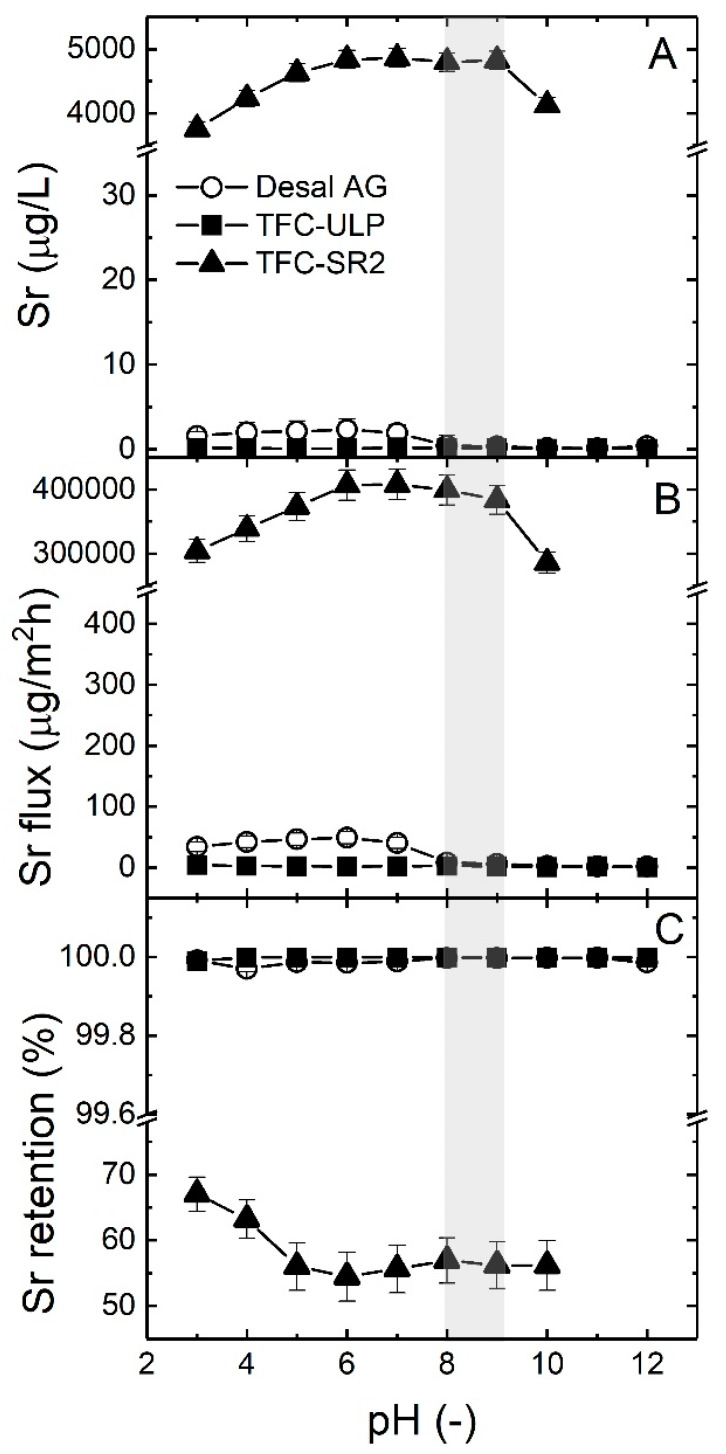
(**A**) Sr permeate concentration, (**B**) Sr flux and (**C**) Sr retention of three membranes as a function of the pH (3–12) of groundwater sample 5. The grey area represents the pH range of groundwater samples 1–5. The applied pressure was 10 bar. Data adapted from [69].

The impact of groundwater pH on Sr removal in real groundwater when using different membranes was investigated by adjusting a range of pH values (3–12) of groundwater prior to the filtration experiments. The Sr flux and retention were calculated for each pH condition. The results are presented in Figure 10.

As shown in Figure 10A,B, TFC-SR2 had the highest Sr permeate concentration with about 4 mg/L (higher than the USEPA guideline of 1.5 mg/L) and Sr flux, while two RO membranes had a very low Sr permeate concentration. This result is in good correlation with much higher Sr retention (almost 100%) of RO membranes compared to the TFC-SR2 membrane (55–67%). The results obtained here confirm the Sr retention trends for different membranes, as shown in Section 3.2.

Sr retention is pH-dependent, although this is more visible when retention is lower. For the Desal AG RO membrane, Sr permeate concentration increased when pH increased from three to six, then decreased sharply when the pH was higher than seven, and then it stayed at a low level, but the change is not significant. This result can probably be explained by the fact that when the pH is higher than seven, RO membranes can easily intercept the dissolved SrCO_3_ (transferred by Sr^2+^, see Figure 8). For TFC-ULP, the pH did not influence Sr retention since almost all Sr was rejected. It is worth noting that for the TFC-SR2 NF membrane, increasing groundwater pH increased Sr permeate concentration initially before leveling out. In the same way, Sr retention significantly decreased and then stayed constant (see Figure 10C). This result may be due to the fact that the membrane surface, which is assumed to be negatively charged, attracted more Sr^2+^ ions with an increase in pH [59].

In summary, alkaline pH conditions could slightly enhance Sr removal of Desal AG RO membrane while such an effect was very weak for the TFC-ULP membrane. Decreased groundwater pH enhanced Sr removal of the TFC-SR2 NF membrane due to increased electrostatic repulsion at the membrane surface or modifications in organic matter interactions. It would be interesting (and indeed this will be investigated in future work) to explore the contribution of such solute–solute interactions on the Sr retention when a membrane retains less Sr (in the case of TFC-SR2). In the next section, the impact of groundwater pH on organic matter removal will be analyzed.

### 3.7. Impact of Groundwater pH on Organic Matter Removal

**Figure 11:**
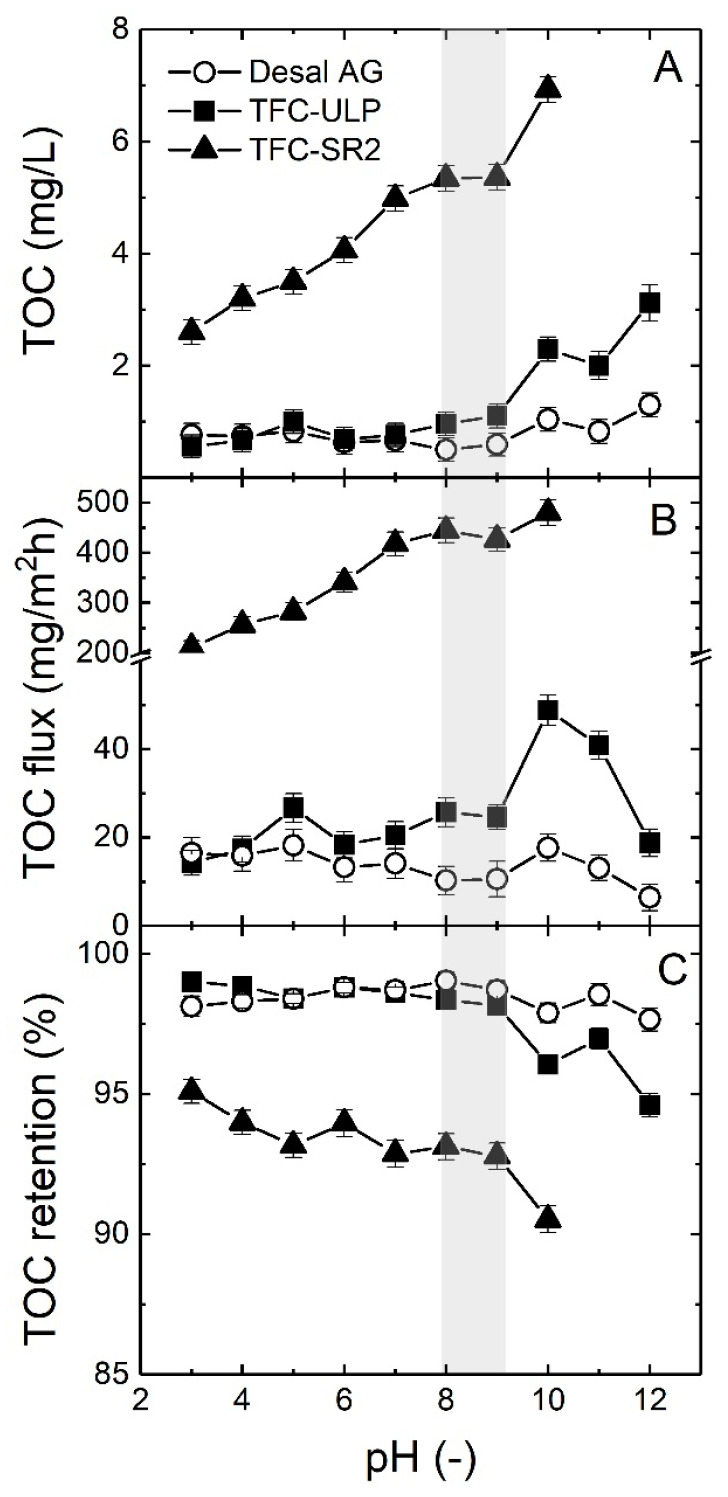
(**A**) TOC permeate concentration, (**B**) TOC flux and (**C**) TOC retention as a function of the pH (3−12) of groundwater sample 5. The grey area is TOC removal at the pH range of groundwater samples 1−5. The applied pressure was 10 bar. Data adapted from [69].

The impact of groundwater pH on organic matter removal by the different NF/RO membranes was investigated by adjusting the pH (3–12) prior to the experiment. The TOC flux and retention at each pH were calculated. The TOC permeate concentration, TOC flux and retention as a function of groundwater pH are presented in Figure 11.

As shown in Figure 11, both TOC permeate concentration, and TOC flux increased, and the TOC retention decreased with an increase in pH, especially for the TFC-ULP and TFC-SR2 membranes, which is unexpected. However, this has been observed in extreme conditions, as reported by Schäfer et al. [72], at high concentrations at the end of a solar day. Due to high CP, the LMW fraction can diffuse through the membrane, and this is more pronounced for the membranes with higher permeability and larger pore size (MWCO).

At high pH, the surface charge of the TFC NF/RO membrane became more negatively charged, and most NOM may become more negative, which would result in high TOC removal due to enhanced charge exclusion [24,71]. Surprisingly, the results show the opposite trend. This result can be attributed to the fact that the organic matter concentration at the membrane surface may enhance the diffusion of organic matter, preferentially LMW compounds [74,76]. This behavior is especially pronounced for the TFC-SR2 NF membrane, which will experience very high concentration polarization due to the high recovery and thus a high organic matter concentration at the membrane surface. The larger pore size (less size exclusion) will allow more small organic molecules to diffuse [76]. This could explain why more NOM could pass through the membrane resulting in a decrease in TOC retention.

The TFC-SR2 NF membrane had a lower TOC retention (90–95%) than the two other RO membranes (95–99%) as expected due to its larger MWCO (see Table 2), resulting in more dissolved NOM passing through the membrane. Compared to RO membranes, the TOC removal of the TFC-SR2 NF membrane also seemed to be more influenced by increasing pH, as shown in Figure 11, which is probably due to the characteristics of the NF membrane being more sensitive to pH than the RO membranes. 

In summary, acidic and neutral pH conditions of groundwater enhanced TOC removal, and the RO membranes had higher TOC retention than the NF membrane. It appears from this work that the Sr–organic matter interaction influences Sr removal, although this comparison could not be made for real water and requires further laboratory investigation.

## 4. Conclusions

In order to understand the removal of natural occurring strontium by the NF/RO system, a natural groundwater source containing high Sr concentration (10.3 mg/L) and organic matter (70.9 mg/L) from Eslalei nearby Lake Manyara in northern Tanzania and a pilot-scale DC operated NF/RO system was selected for experimental investigations. Applied pressure and pH are essential variables in addition to organic matter content and ionic compositions when treating groundwater. Therefore, two sets of experiments (applied pressure 10–15 bar and pH 3–12) with three membranes were carried out. Membrane performance including permeate flux, solute flux and solute removal was examined. 

Increasing the applied pressure (up to 15 bar) enhanced the permeate flux through increased driving force for the filtration process, Sr removal and TOC removal were enhanced by the dilution effect (water flux was higher than solute flux).

By analyzing the organic matter fractions of the groundwater using LC-OCD, it was seen that some LMW organic matter fractions were high. These LMW organics may pass through the RO membrane, resulting in only 98.5% TOC removal. The alkaline pH condition of groundwater caused severe flux decline, likely due to membrane organic fouling and scaling. Slightly enhanced Sr removal of the RO membrane, but weakened TOC removal, was observed under these conditions. In contrast, acidic pH condition enhanced Sr removal for the NF membrane (TFC-SR2), presumably due to an increase in electrostatic repulsion at the membrane surface. Acidic and neutral pH conditions of groundwater enhanced TOC removal. The TFC-ULP RO membrane had higher permeate flux and slightly higher Sr retention than that of the Desal AG RO membrane, while Desal AG had slightly higher TOC retention than TFC-ULP. 

It is worth noting that even though the TFC-SR2 NF membrane could not achieve the USEPA guideline of Sr in this case (high Sr content), and cannot achieve adequate desalination for such water, an increase in pH caused a more pronounced decrease in Sr and TOC retention than RO membranes, which means TFC-SR2 could be a suitable membrane for investigating the Sr–organic matter transport mechanisms by NF membrane determined by using real groundwater. Additionally, the interaction of Sr and organic matter requires further investigation.

The appropriately high applied pressure and acidic pH condition of groundwater are recommended to achieve better Sr removal by NF/RO membranes. The required pressure is case-specific, depending on feed water quality, membrane choice, containment removal, and system design. Therefore, a pilot filtration test of the specific feed solution under different applied pressures should be performed to determine the required applied pressure.

## Figures and Tables

**Table 1:** Physicochemical properties of calcium, strontium and barium (data adapted from [3,9,15,16,17]).

Element	Atomic Number	Relative Molar Mass (g/mol)	Melting Point (°C)	Ionic Radius (nm)	Hydrated Radius (nm)	Solubility (g/L)			
						Hydroxide	Chloride	Carbonate	Sulfate
Calcium	20	40.08	842	0.100	0.412	1.31	813.85	0.013	1.11
Strontium	38	87.62	777	0.125	0.412	10.45	554.82	0.006	0.114
Barium	56	137.33	727	0.136	0.404	46.64	372.50	0.009	0.003

**Table 2:** Characteristics of the selected nanofiltration/reverse osmosis (NF/RO) membranes.

Membrane Model	MWCO (g/mol)	Estimated Pore Radius (nm)	Isoelectric Point (IEP)	Salt Retention (%) *	Pure Water Permeability (L/m^2^h·bar)	pH Range *
Desal AG-2540 (RO)	145 [58]	0.26 [58]	-	2 g/L NaCl: 99.5	3.2 [58]	2–11.5
TFC ULP-2540 (RO)	<180 [61]	<0.64 [61]	<3 [60]	2 g/L NaCl: 99.0	4.4 *	2–12
TFC SR2-2540 (NF)	460 [59]	0.52 [59]	4.25 [59]	5 g/L MgSO_4_: 95.0	21.3 [62]	3–11

* Reported by the manufacturer.

**Table 3:** The summary of the experimental protocol using pilot-scale GE Osmonics E2-2535TM system.

No	Step	Operation	Justification
1	System checking and cleaning	The tightness of all tubing connections was checked. The system was cleaned with DI water.	To ensure components are well connected and the system is clean
2	System start-up	First the booster pump; then the high-pressure pump was started to pump the feed water through the membrane modules.	To start the filtration experiment with the groundwater sample
3	Operating pressure setting	The pressure was adjusted for operating pressure experiments with the concentrate valve to vary from 10 to 15 bar. For pH experiments, it was kept at 10 bar. Permeate and concentrate were recirculated.	To investigate the effect of operating pressure and pH of samples on Sr and TOC removal by NF/RO. Filtration was performed until a steady state was reached.
4	Samples collecting	The permeate samples were collected after the steady-state was reached.	To measure the Sr and TOC concentration
5	System cleaning	2% citric acid and DI water (conductivity < 1 μs/cm) were used for system cleaning.	To remove any accumulated foulants or scalants from the membrane surface and restore system performance

**Table 4:** Water quality of the groundwater samples in the Esilalei area (samples 1–4 were analyzed at Engler-Bunte Institute (EBI-KIT), and sample 5 was analyzed at the Beijing University of Chemical Technology (BUCT). Sample 5 was used as the feed solution in this study. Water quality variation occurs with location, season (in particular, rainfall) and probably extraction method/volume.

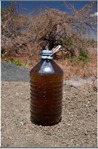	Unit	Sample 1	Sample 2	Sample 3	Sample 4	Sample 5	WHO Guideline [11]
Electrical conductivity	μS/cm	16,150	27,100	27,600	28,500	14,340	400
pH	-	8.99	8.08	8.49	8.18	8.98	6.5–8.5
Turbidity	NTU	38.96	14.55	1.14	15.79	11.36	5
Sodium	mg/L	4730.9	8472.5	9012.5	7256.7	4240.5	200
Potassium	mg/L	91.9	272.0	288.5	271.0	123.4	20
Calcium	mg/L	10.1	10.1	128.3	122.8	210.5	100
Magnesium	mg/L	8.0	605.4	638.9	505.2	327.2	50
Strontium	mg/L	1.2	8.2	8.0	10.4	10.3	-
Chloride	mg/L	486.1	569.0	554.0	593.1	587.2	250
Fluoride	mg/L	56.6	9.2	8.9	4.6	6.2	1.5
Sulfur	mg/L	1974.3	5860.0	6204.4	6391.5	2473.4	-
Inorganic carbon	mg/L	908.0	420.2	580.8	960.0	454.0	-
TOC	mg/L	58.2	57.2	46.6	107.0	70.9	-
